# Unusual Presentation of Chronic Eosinophilic Pneumonia with “Reversed Halo Sign”: A Case Report

**DOI:** 10.5812/iranjradiol.7891

**Published:** 2014-05-15

**Authors:** Mahdia Gholamnejad, Nader Rezaie

**Affiliations:** 1 Department of Pulmonology, Imam Khomeini Hospital, Urmia University of Medical Sciences, Urmia, Iran; 2 Department of Pulmonology, Firouzgar Hospital, Iran University of Medical Sciences, Tehran, Iran

**Keywords:** Reversed Halo Sign, Carrington Syndrome, Tomography, X-Ray Computed

## Abstract

The reversed halo sign (RHS) may sometimes be seen in patients with cryptogenic organizing pneumonia (COP), but is rarely associated with other diseases. Herein, we present a case of a 21-year-old woman with chronic eosinophilic pneumonia, with high resolution computed tomography (HRCT) finding of RHS. This is an unusual and rare presentation of chronic eosinophilic pneumonia.

## 1.Introduction

The reversed halo sign (RHS) is defined as a focal, rounded area of ground-glass opacity surrounded by a complete or nearly complete ring of consolidation, as demonstrated on HRCT scan of the chest (1). RHS has been reported in association with a wide variety of clinical entities, including infectious and noninfectious diseases (2-4). Chronic eosinophilic pneumonia (CEP) is an idiopathic condition characterized by chronic infiltration of lung with eosinophils. The characteristic radiographic finding of CEP consists of peripheral nonsegmental areas of consolidation, the photographic negative of pulmonary edema involving mainly the upper lobes (5). Hereby, we present an unusual case of CEP with an RHS.

## 2. Case Presentation

A 21-year-old nonsmoking woman was admitted to our hospital with a 3-month history of progressive dyspnea and cough. The patient also complained of malaise, anorexia and weight loss. The initial diagnosis by her physician was “asthma”, based on clinical finding and spirometry described as a severe obstructive pattern. She received inhaled corticosteroid and β_2_- agonist with no significant improvement. She had no history of any other drugs or alternative medicines. On physical examination, the patient was ill appearing, with a frequent dry cough. Coarse crackles and generalized wheezing were detected on lung auscultation. Laboratory finding revealed severe eosinophilia comprising 21.2% of the total peripheral white blood cell count (2450 /µL) and an elevated serum level of erythrocyte sedimentation rate (ESR) and immunoglobulin E (IgE). There were no fecal findings of parasitic infestation. Tests for antinuclear antibody (ANA) and anti neutrophil cytoplasmic antibody (ANCA) were negative.

**Figure 1.**
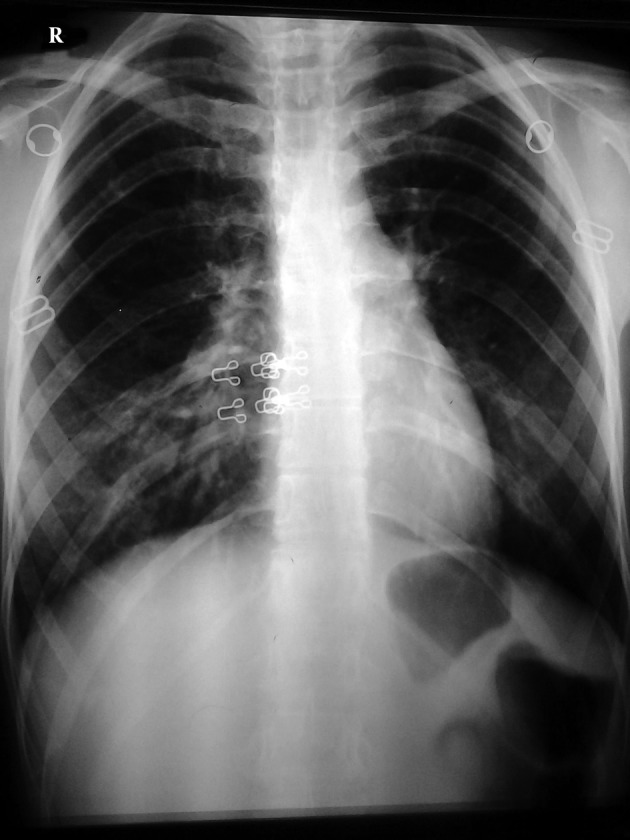
Posteroanterior chest radiograph shows patchy ill-defined infiltrates

**Figure 2.**
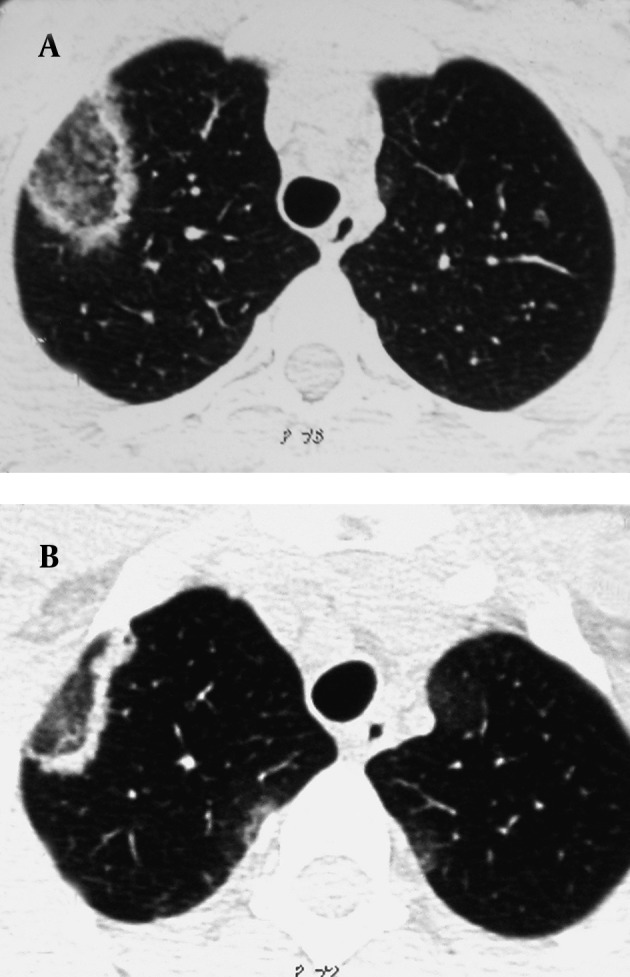
A, B) Axial CT images at the level of great vessels show opacity with RHS appearance

**Figure 3.**
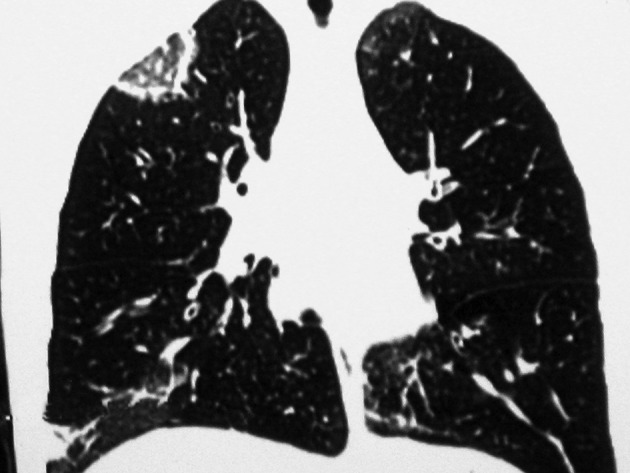
Coronal view shows RHS in the right upper lobe and an ill-defined opacity in the lower lobe

Chest radiography demonstrated bilateral patchy ill-defined infiltrations (Figure 1). An HRCT scan of the chest showed RHS in the right upper lobe and few ill-defined peripheral air-space consolidations (Figures 2 and 3). Mediastinal or pleural abnormality was not seen.

**Figure 4.**
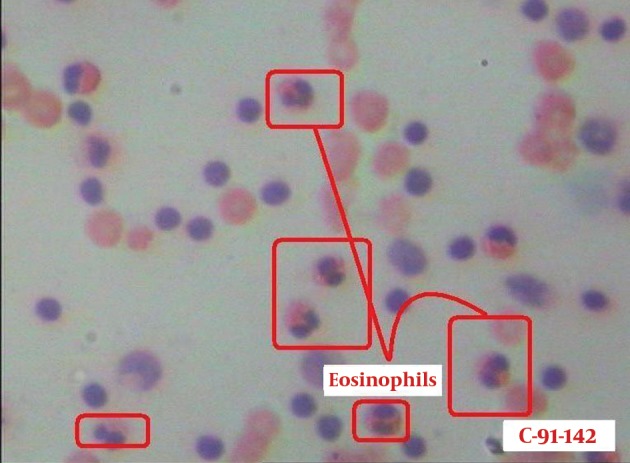
BALF smear shows many eosinophils

**Figure 5.**
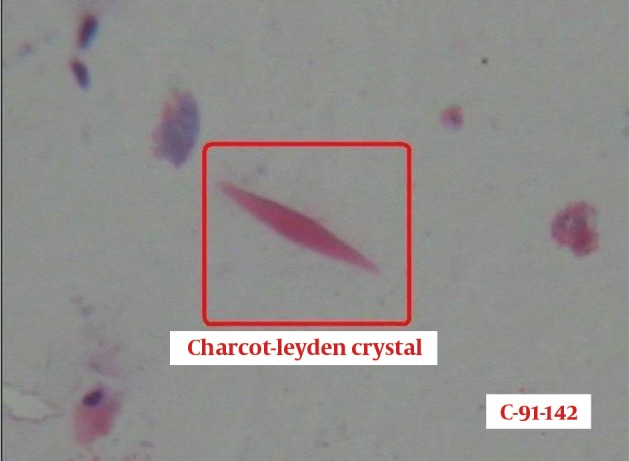
BALF smear shows Charcot-Leyden crystals

**Figure 6.**
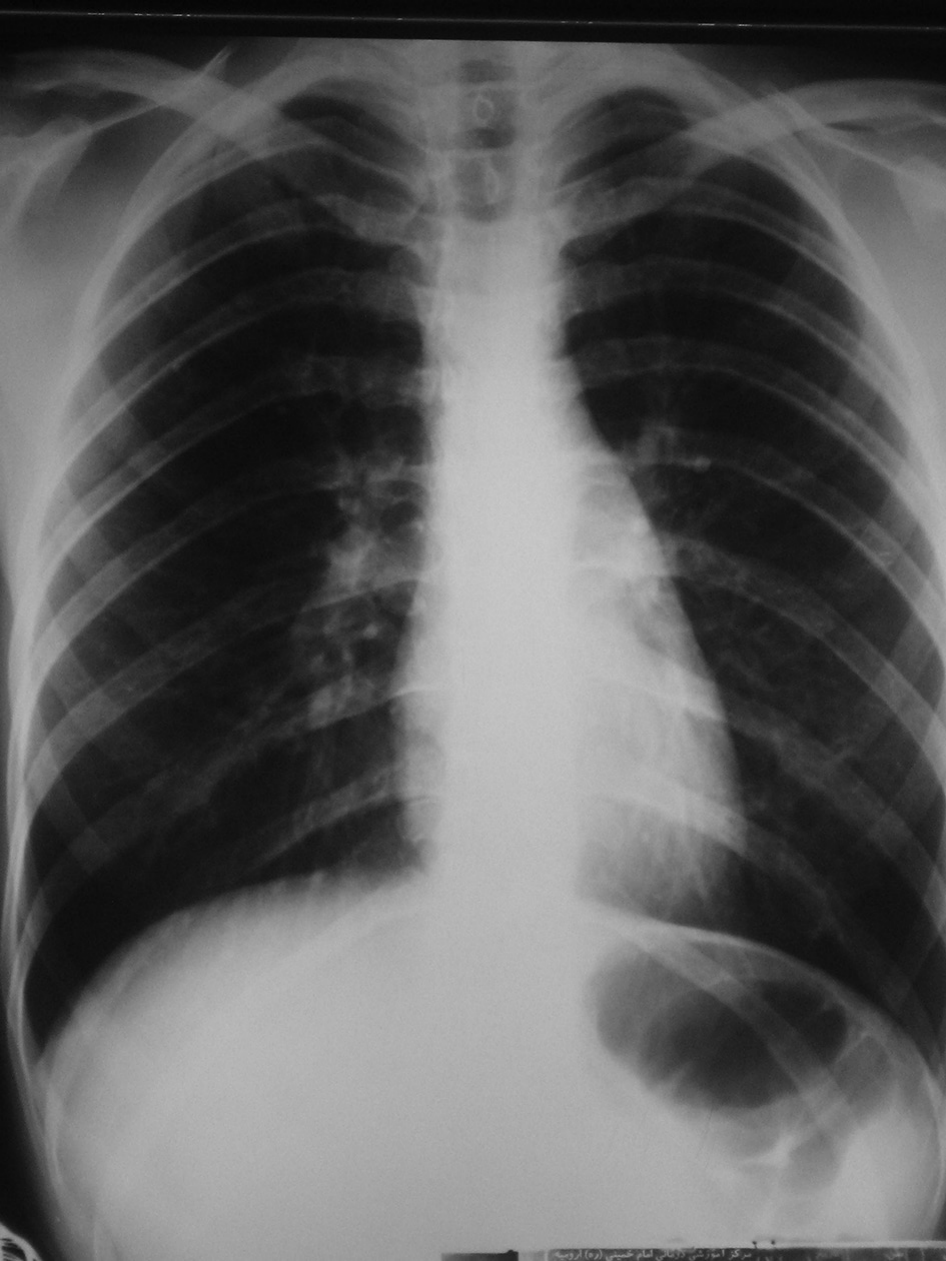
Posteroanterior chest radiograph shortly after treatment shows improvement of abnormalities

Bronchoscopy was done and bronchoalveolar lavage fluid (BALF) analysis revealed a marked increase in the number of cells with a high proportion of eosinophils (45.4%) (Figure 4). Charcot-Leyden crystals were seen indicating eosinophil activation (Figure 5). There was no evidence of bacterial pathogens or malignant cells. Cytomorphology of BALF was not in favor of extrinsic allergic alveolitis or cryptogenic organizing pneumonia (COP). With the diagnosis of idiopathic eosinophilic pneumonia, oral prednisolone (0.5/kg/day) and inhaled corticosteroid was initiated. One week after treatment, the symptoms improved dramatically and radiologic abnormalities completely resolved (Figure 6). Five months after starting treatment and on tapering the dose of corticosteroid, the patient was free of symptoms and chest imaging was normal.

## 3. Discussion

RHS was described initially in patients with COP and was considered to be an HRCT scan finding specific for this disorder (3). Other studies have shown the presence of this sign in a wide spectrum of conditions, including infectious diseases such as tuberculosis (6), zygomycosis (7), and pneumocystis carinii (8), and noninfectious diseases such as secondary organizing pneumonia (9), granulomatosis with polyangiitis (10), and sarcoidosis (11). CEP is a cryptogenic form of eosinophilic lung disease with characteristic clinicopathological features. Imaging of CEP is characteristic, although many of the features overlap with those found in COP. Peripheral opacities in chest x-ray are described in many cases, and are migratory in a quarter of the cases (12). The classic pattern of photographic negative or reversal of the shadows usually seen in pulmonary edema, highly evocative of CEP, is seen in only one fourth of the patients (13); chest radiography of our patient did not show this finding. HRCT has further allowed description of the characteristic features of CEP. Characteristic opacities are predominant in the upper lobes and are peripheral with generally coexisting ground-glass and air-space consolidations (5).

Although the radiologic manifestations of our case show some similarity with typical radiologic manifestations of CEP, to our knowledge, CEP has never been reported in connection with the RHS. In histopathologic studies of eosinophilic pneumonia, organization of the alveolar inflammatory exudate is a rather common finding (14), suggesting some possible overlap between CEP and COP. This possible pathologic overlap may explain our radiologic findings. However, in CEP, intraluminal organization in the distal air spaces is only sparse and never prominent (14). Although in this patient, lung biopsy was not performed (lung biopsy is seldom necessary), laboratory findings and BALF analysis results were incompatible with the diagnosis of COP.

The findings of this report reinforce the nonspecific nature of RHS in lung disorders. This is likely because there are a number of different mechanisms that can cause this characteristic pattern. Although the presence of RHS may help to narrow the range of diseases considered in making the differential diagnosis, final diagnosis should be based on the clinical scenario and the presence of laboratory and additional disease specific CT scan findings.
